# Evaluation of a short dynamic ^18^F-fluoride PET/CT scanning method to assess bone metabolic activity in spinal orthopedics

**DOI:** 10.1007/s12149-015-1008-0

**Published:** 2015-08-05

**Authors:** Marloes J. M. Peters, Roel Wierts, Elisabeth M. C. Jutten, Servé G. E. A. Halders, Paul C. P. H. Willems, Boudewijn Brans

**Affiliations:** Department of Orthopedic Surgery, Maastricht University Medical Center, Postbox 5800, 6202 AZ Maastricht, The Netherlands; Department of Nuclear Medicine, Maastricht University Medical Center, Maastricht, The Netherlands

**Keywords:** ^18^F-fluoride PET/CT, SUV, Kinetic modeling, Lumbar spine, Spinal fusion

## Abstract

**Objective:**

A complication after spinal fusion surgery is pseudarthrosis, but its radiological diagnosis is of limited value. ^18^F-fluoride PET with its ability to assess bone metabolism activity could be of value. The goal of this study was to assess the clinical feasibility of calculating the static standardized uptake value (SUV) from a short dynamic scan without the use of blood sampling, thereby obtaining all dynamic and static parameters in a scan of only 30 min. This approach was tested on a retrospective patient population with persisting pain after spinal fusion surgery.

**Methods:**

In 16 patients, SUVs (SUV_max_, SUV_mean_) and kinetic parameters (*K*_1_, *k*_2_, *k*_3_, *v*_b_, *K*_i,NLR_, *K*_1_/*k*_2_, *k*_3_/(*k*_2_ + *k*_3_), K_*i,*patlak_) were derived from static and dynamic PET/CT scans of operated and control regions of the spine, after intravenous administration of 156–214 MBq ^18^F-fluoride. Parameter differences between control and operated regions, as well as between pseudarthrosis and fused segments were evaluated. SUV_mean_ at 30 and 60 min was calculated from kinetic parameters obtained from the dynamic data set (SUV_mean,2TCM_). Agreement between measured and calculated SUVs was evaluated through Bland–Altman plots.

**Results:**

Overall, statistically significant differences between control and operated regions were observed for SUV_max_, SUV_mean_, K_*i,*NLR_, K_*i,*patlak_, *K*_1_/*k*_2_ and *k*_3_/(*k*_2_ + *k*_3_). Diagnostic CT showed pseudarthrosis in 6/16 patients, while in 10/16 patients, segments were fused. Of all parameters, only those regarding the incorporation of bone [*K*_i,NLR_, *K*_i,patlak_, *k*_3_/(*k*_2_ + *k*_3_)] differed statistically significant in the intervertebral disc space between the pseudarthrosis and fused patients group. The mean values of the patient-specific blood clearance rate $\tau^{*}$ differed statistically significant between the pseudarthrosis and the fusion group, with a *p* value of 0.011. This may correspond with the lack of statistical significance of the SUV values between pseudarthrosis and fused patients. Bland–Altman plots show that calculated SUV_mean,2TCM_ values corresponded well with the measured SUV_mean_ values.

**Conclusion:**

This study shows the feasibility of a 30-min dynamic ^18^F-fluoride PET/CT scanning and this may provide dynamic parameters clinically relevant to the diagnosis of pseudarthrosis.

## Introduction

Low back pain is a major global health and economic problem [1–3], with a 1-year prevalence ranging from 22 to 65 % and lifetime prevalence of up to 84 % [4]. The direct costs, including patient care, medical procedures and medication are acceptable, however, the yearly indirect costs caused by absence from work and early retirement are manifold [2].

Low back pain is mainly caused by degenerative disorders of the spine, such as spondylolisthesis, degenerative scoliosis, degenerative disc disease, or recurrent disc herniations [5, 6]. If conservative measures such as intensive exercise therapy, pain medication or brace immobilization fail, spinal fusion is considered. In at least 15 % of primary lumbar fusions, pseudarthrosis occurs instead of bony fusion [7, 8]. Pseudarthrosis is defined as the absence of solid fusion (nonunion) 1 year after the operation, and is typically associated with axial or radicular pain [7, 9]. Although solid fusion is not required for pain relief [10], pseudarthrosis in general, even without clinical symptoms, increases the risk of clinical failure, late deformity, neurological symptoms and pain [11].

Surgical exploration remains the gold standard for diagnosing pseudarthrosis [7, 9, 12–15]. Current non-invasive, imaging assessment of pseudarthrosis in patients with persistent or recurrent symptoms after spinal fusion includes plain radiography, flexion–extension radiography, ultrasound, bone scintigraphy, computed tomography (CT) and magnetic resonance imaging (MRI). Most radiological modalities aim at the detection of well-established pseudarthrosis by looking at anatomical signs of bony connection between the vertebrae. In contrast, single-photon emission computed tomography (SPECT) and positron emission tomography (PET) are 3D functional imaging modalities looking at biological processes underlying the process of fusion. Therefore, these imaging techniques may detect an evolving pseudarthrosis. A few studies have reported on the use of PET/CT scanning for the detection of union/pseudarthrosis after spinal fusion and have indicated the value of ^18^F-fluoride PET/CT scanning in symptomatic patients [16–19].

These studies have analyzed the PET images by calculating the standardized uptake value (SUV) to assess bone metabolism. SUV is a valuable tool in clinical practice, easy and fast to use, and can provide reproducible results. However, SUV is dependent on the time after injection [20], and the rate of clearance of the radiotracer from the arterial blood [21]. For ^18^F-fluoride, Blake [22] has shown that SUV is not optimal in patients with disorders or drugs having an effect on the whole skeleton bone metabolism due to increased blood clearance. Full pharmacokinetic analysis, yielding the fluoride bone influx rate *K*_i_ (*K*_i,NLR_ and *K*_i,patlak_) based on the Hawkins model [23], is not dependent on time after injection nor on blood clearance rate. So far, the benefits that dynamic scanning yields have been outweighed by the practical use and ease of static scanning. Siddique has stated that it is possible to calculate the dynamic parameter *K*_i_ from a static scan in combination with several venous blood samples, obviating the need to make dynamic scans [21]. However, this excludes calculation of the individual dynamic parameters that *K*_i_ is composed of, while the significance of these additional parameters has not been fully explored for this patient population.

The goal of this study was to assess the clinical feasibility of obtaining dynamic and static parameters from a 30-min scan without the use of blood sampling and compare these parameters in a retrospective patient population with persisting pain after spinal fusion surgery.

## Materials and methods

### Patients

A cohort of 16 patients was enrolled in this study between June 2008 and February 2015. Patients who underwent posterior lumbar interbody fusion (PLIF) surgery for the indication 1–2 grade degenerative spondylolisthesis, and who suffered from persisting or recurrent low back pain after the procedure without an obvious clinical or radiological explanation were included in the study. The patient group consisted of 11 female and 5 male patients, with a mean age at surgery of 44.9 years (range 26–64 years) and a body mass index (BMI) of 29.5 kg/m^2^ (range 19.3–44.6 kg/m^2^). Patients were operated on level L3–L4 (*n* = 2), L4–L5 (*n* = 4) or L5–S1 (*n* = 10). The time interval between fusion surgery and the PET/CT examination was 4–75 months (mean 22 months, median 17 months). One patient underwent PLIF surgery at two levels. Therefore, the total number of operated levels to be analyzed was 17. This study was performed in accordance with the Helsinki Declaration of 1975, as revised in 2013, and was part of a protocol accepted by the medical ethical committee of the Maastricht University Medical Center (NL.32881.068.11) in which patients gave their written and informed consent.

### Posterior lumbar interbody fusion (PLIF), surgical technique

Under general anesthesia and in a prone position, the vertebral arches of the intended levels were identified under fluoroscopic control and exposed by an open posterior lumbar approach. Nerve roots were decompressed by laminectomy and the intervertebral disc was excised. After thorough cleansing of the endplates, two 10–12 mm intervertebral cages (Capstone^®^ PEEK, Medtronic, Memphis, USA), filled with autologous bone from the vertebral lamina, were inserted into the disc space, right and left of the midline. Additionally, the remaining disc space was packed with autologous bone chips from the laminectomy. Next, the upper and lower vertebrae were fixed by 4 transpedicular screws with titanium rods (CD Legacy^®^, Medtronic, Memphis, USA) for primary stabilization.

### ^18^F-fluoride PET/CT scans

The PET and CT images were acquired with an integrated PET/CT scanner (Gemini TF PET-CT, Philips, The Netherlands). First, a low-dose CT acquisition (120 kV, 30 mAs, slice thickness 4 mm) used for localization purposes and attenuation correction of the PET images was made. Immediately after intravenous injection of 156-214 MBq (mean 188 MBq; median 186 MBq) Na-(^18^F)-fluoride, the dynamic scanning started which involved a three-dimensional 30-min list mode PET scan of the operated segment in an 18-cm axial field of view. This list mode scan was rebinned into the following consecutive time frames: 6 × 5, 3 × 10, 9 × 60, 10 × 120 s. Static scanning involved a low-dose CT acquisition (parameters as in the dynamic case) followed by a conventional PET scan 60 min after injection, covering the whole lumbosacral spine, acquired by two bed positions of 5 min each. This was immediately followed by a high-dose, non-contrast enhanced CT scan (64-slice helical, 120 kV, 250 mAs, slice 1 mm with increment of 0.8 mm) of the fusion region. Standard filtered backprojection CT reconstruction was performed. PET images were reconstructed into both non-attenuated and CT-based attenuated images using the standard blob-os-TF reconstruction algorithm. Images were viewed on clinical software (EBW, Philips, The Netherlands) and further analyzed by a research tool (PMOD 3.0, PMOD Technologies Ltd, Zürich).

### Analysis of ^18^F-fluoride PET/CT scans

Twelve parameters were derived from the static and dynamic PET scans. The mean and maximum SUV at 30 min were calculated from the last frame of the dynamic scan (SUV_mean30_ and SUV_max30_). The mean and maximum SUV at 60 min were calculated from the static scan (SUV_mean60_ and SUV_max60_). The analysis of the dynamic scans was based on the 2 tissue compartment model (2TCM) [23]. Through nonlinear regression (NLR) analysis, *K*_1_, *k*_2_, *k*_3_, *v*_b_, *K*_i,NLR_, *K*_1_/*k*_2_ and *k*_3_/(*k*_2_ + *k*_3_) were calculated from the dynamic scan. *K*_i,patlak_ was calculated from the dynamic scan through Patlak graphical analysis [24, 25].

**Fig. 1:**
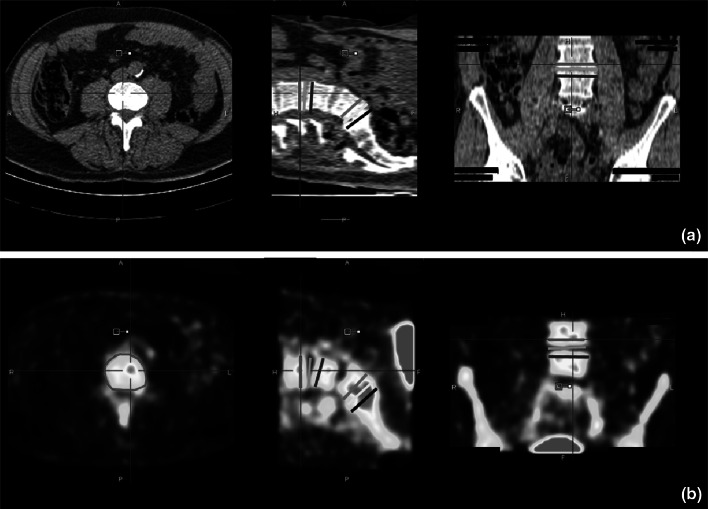
ROI definition. **a** An axial, sagittal and coronal CT image (from *left* to *right*) of the lower spine after PLIF. Three ROIs were drawn in the operated segment: the lower endplate of the cranial vertebra (*pink*), the intervertebral disc (*yellow*), the upper endplate of the caudal vertebra (*black*). The same three ROIs were drawn in a normal reference segment 2 levels above the operated segment (*red*, *green* and *blue*, respectively). **b** The six ROIs were transferred to the co-registered PET image (axial, sagittal and coronal views from *left* to *right*)

The twelve parameters were calculated based on a region of interest (ROI) approach. In each CT scan, 6 ellipsoid-shaped ROIs were manually drawn following the contours of the vertebrae (slice thickness 4 mm, short axis range 40–50 mm, long axis range 55–65 mm), including the intervertebral disc space and upper and lower endplates of the operated segment as well as of a control segment 2 levels higher (Fig. 1a). These ROIs were transferred to the co-registered attenuation-corrected PET image (Fig. 1b).

1. SUV was obtained by correcting the radioactivity concentration measured by the PET scanner [*A* (kBq/ml)] for the injected dose of ^18^F-fluoride [ID (MBq)] and the body weight of the patient [*m* (kg)] according to (1).
  1 $${\text{SUV}} = \frac{A}{{{\text{ID}}/m}}$$
2. For dynamic analysis, the arterial blood input function and the tissue time–activity curves (TACs) were needed. The same 6 ROIs that were used in static analysis were applied to the dynamic frames to generate the TACs. The arterial blood input function was determined by means of an image-derived input function (IDIF) obtained via a thresholding method. The frames of the dynamic PET scan that showed a clear bolus (2–4 frames early in the dynamic scan) were summed. In the summed image, a 75 % threshold was applied to a box placed manually around the abdominal aorta, to obtain a volume of interest (VOI). By applying the VOI to all dynamic frames, the IDIF was generated.
  The IDIF and ROI TACs were fitted to the 2TCM using a nonlinear regression algorithm and a Patlak algorithm (PMOD 3.0, PMOD Technologies Ltd, Zürich) estimating the kinetic parameters *K*_1_, *k*_2_, *k*_3_, *v*_b_ and *K*_i,patlak_. The parameter *k*_4_ was assumed to be negligible and set to 0.
  The fluoride bone influx rate, *K*_i_ (previously referred to in literature as *K*_bone_), represents the net uptake rate of ^18^F in the bone mineral, calculated as a combination of the rate constants (2). *K*_1_ is correlated to bone perfusion [26]. Also calculated was *k*_3_/(*k*_2_ + *k*_3_), which represents the fraction of tracer entering the tissue compartment that undergoes specific binding to the bone mineral [26] and *K*_1_/*k*_2_, which represents the volume of distribution of tracer in the unbound pool [27].
  2 $$K_{\text{i, NLR}} = \frac{{K_{1} \times k_{3} }}{{k_{2} + k_{3} }}$$
3. The relationship between SUV and *K*_i,NLR_ can be written as (3), which was derived using the analytical solution of the 2TCM for the time-dependent tissue radioactivity concentration. In which, *f*_b_ represents the blood fraction (L/kg) defined as the ratio of total blood volume to body mass and *τ* (Bq s/Bq) is the blood fractional residence time in a region, defined as the mean time that an administered substance spends in that region [28]. The full derivation can be found in “Appendix”.
  3 $$\mathop {\lim }\limits_{t \to \infty } SUV_{{ 2 {\text{TCM}}}} \approx K_{\text{i, NLR}} \times \frac{\tau }{{f_{\text{b}} }} = K_{\text{i, NLR}} \times \tau^{*}$$
  In (3), the factor $\tau^{*}$ (Bq s/Bq) is defined as the blood residence time normalized to the blood fraction. The residence time as well as the blood fraction are patient-specific factors that are not directly related to metabolic bone activity at a specific site of interest but do have an effect on SUV. This relationship together with the *K*_i,NLR_ values was used to calculate SUV at 30 and 60 min from the 30-min dynamic scan (SUV_mean30,2TCM_ and SUV_mean60,2TCM_, respectively). Moreover, the factor $\tau^{*}$ was calculated for each patient to evaluate the inter-subject variability that this factor introduces to the SUV. Based on the CT scan, patients were divided into two categories. Patients who had no signs of bony bridging between the two operated vertebrae were categorized as pseudarthrosis. Patients with bony bridges were categorized as fused.

### Statistical analysis

Statistical evaluation was performed using IBM SPSS Statistics for Windows, Version 20.0 (Armonk, NY: IBM Corporation). To test the data for normality of distribution, the Shapiro–Wilk test was used. The Pearson correlation test was used to examine the correlation between the different methods. The goodness of the fit was determined through calculation of *R*^2^. The differences between the control and the operated regions, and between the pseudarthrosis and fused patients were compared using the Wilcoxon signed-rank test. The magnitude of the observed differences between operated and control regions was evaluated by calculation of the Cohen’s *d* effect size, which is defined as the difference between the two means of the subgroups divided by the standard deviation of the complete data set. In practice, the higher (positive or negative) the value of Cohen’s *d* effect size, the larger the difference is. To determine whether the calculated SUV agreed with the measured SUV, Bland–Altman plots were evaluated [29]. *p* values smaller than or equal to 0.05 were considered statistically significant.

## Results

Table 1 summarizes the fluoride bone metabolic values, i.e., SUV, Patlak and NLR rate constants for the control and the operated regions. As can be seen, statistically significant differences between control and operated regions (upper, lower endplate and intervertebral) were found for most of the obtained parameters, although not for the individual rate constants *K*_1_, *k*_2_, *k*_3_ and *v*_b_. The highest statistical significance values were found for SUV_mean60_ (0.001–0.002), *K*_i,patlak_ (0.001–0.003), *K*_i,NLR_, *K*_1_/*k*_2_ (0.004–0.03) and *k*_3_/(*k*_2_ + *k*_3_) (0.003–0.02).

**Table 1:** Parameter values of control and operated regions

Parameter	Region of interest	Control regions^a^	Operated regions^a^	*p* value^b^	Effect size
SUV_mean30_ (–)	UE	5.0 (1.2)	7.7 (2.2)	0.002	1.6
	IDS	2.3 (0.74)	7.0 (2.1)	0.001	3.6
	LE	5.1 (1.1)	7.8 (2.0)	0.001	1.3
SUV_max30_ (–)	UE	6.7 (1.7)	11 (3.3)	0.002	1.8
	IDS	3.6 (1.5)	11 (3.1)	0.001	3.3
	LE	7.2 (1.9)	11 (3.2)	0.001	1.6
SUV_mean60_ (–)	UE	6.2 (1.1)	8.6 (2.3)	0.002	1.6
	IDS	2.6 (0.83)	7.3 (2.1)	0.001	3.3
	LE	6.4 (1.2)	8.9 (2.5)	0.002	1.3
SUV_max60_ (–)	UE	8.4 (1.6)	13 (3.8)	0.001	1.5
	IDS	4.4 (2.5)	12 (3.2)	0.001	3.6
	LE	9.1 (2.8)	14 (4.9)	0.005	1.3
*K*_i,patlak_ (min^−1^)	UE	0.053 (0.012)	0.081 (0.023)	0.003	1.7
	IDS	0.020 (0.0072)	0.075 (0.022)	0.001	3.4
	LE	0.050 (0.011)	0.084 (0.021)	0.001	1.9
*K*_i,NLR_ (min^−1^)	UE	0.055 (0.012)	0.079 (0.020)	0.002	1.8
	IDS	0.022 (0.0064)	0.073 (0.025)	0.001	3.5
	LE	0.053 (0.011)	0.076 (0.029)	0.011	1.8
*K*_1_ (ml g^−1^ min^−1^)	UE	0.23 (0.12)	0.19 (0.027)	0.463	−0.29
	IDS	0.14 (0.065)	0.18 (0.037)	0.022	0.70
	LE	0.20 (0.067)	0.20 (0.076)	0.594	−0.065
*k*_2_ (min^−1^)	UE	1.0 (1.7)	0.23 (0.21)	0.019	−0.63
	IDS	0.41 (0.25)	0.27 (0.25)	0.14	−0.61
	LE	0.27 (0.13)	0.32 (0.64)	0.056	0.12
*k*_3_ (min^−1^)	UE	0.21 (0.19)	0.14 (0.13)	0.055	−0.33
	IDS	0.076 (0.039)	0.16 (0.15)	0.026	0.87
	LE	0.098 (0.041)	0.15 (0.15)	0.363	0.51
*v*_b_ (–)	UE	0.027 (0.016)	0.041 (0.017)	0.003	0.99
	IDS	0.032 (0.011)	0.040 (0.017)	0.022	0.50
	LE	0.041 (0.019)	0.040 (0.018)	0.975	−0.23
*K*_1_/*k*_2_ (ml g^−1^)	UE	0.56 (0.33)	2.2 (2.4)	0.004	0.84
	IDS	0.42 (0.21)	1.8 (2.0)	0.003	1.0
	LE	0.89 (0.32)	3.2 (5.5)	0.03	0.47
*k*_3_/(*k*_2_ + *k*_3_) (–)	UE	0.29 (0.14)	0.41 (0.099)	0.003	1.3
	IDS	0.18 (0.088)	0.40 (0.14)	0.004	2.5
	LE	0.29 (0.089)	0.41 (0.20)	0.016	1.2

*UE* upper endplate, *IDS* intervertebral disc space, *LE* lower endplate

^a^Values as mean (SD in brackets), 2 significant digits

^b^The *p* value indicates the statistical difference between the operated and control region

When comparing the Cohen’s *d* effect size of the operated to the control regions, parameters concerning bone metabolism (SUV_mean60_, SUV_max60_, *K*_i,patlak_ and *K*_i,NLR_) showed larger differences for the intervertebral disc space (around 3.5) than for the endplates (around 1.5). This corresponded to the increase of bone turnover in the fusion region in contrast to the non-osseous normal intervertebral disc space. Equation (2) shows that bone metabolism parameter *K*_i,NLR_ consists of a part related to bone blood perfusion, *K*_1_, and a part related to bone incorporation *k*_3_/(*k*_2_ + *k*_3_). As can be seen in Table 1, looking at the intervertebral values, the effect size for *k*_3_/(*k*_2_ + *k*_3_) was 2.5, whereas the effect size for *K*_1_ was only 0.70, indicating that the high effect size of *K*_i,NLR_, was mainly caused by a higher amount of bone incorporation and not due to an increase in *K*_1_.

**Fig. 2:**
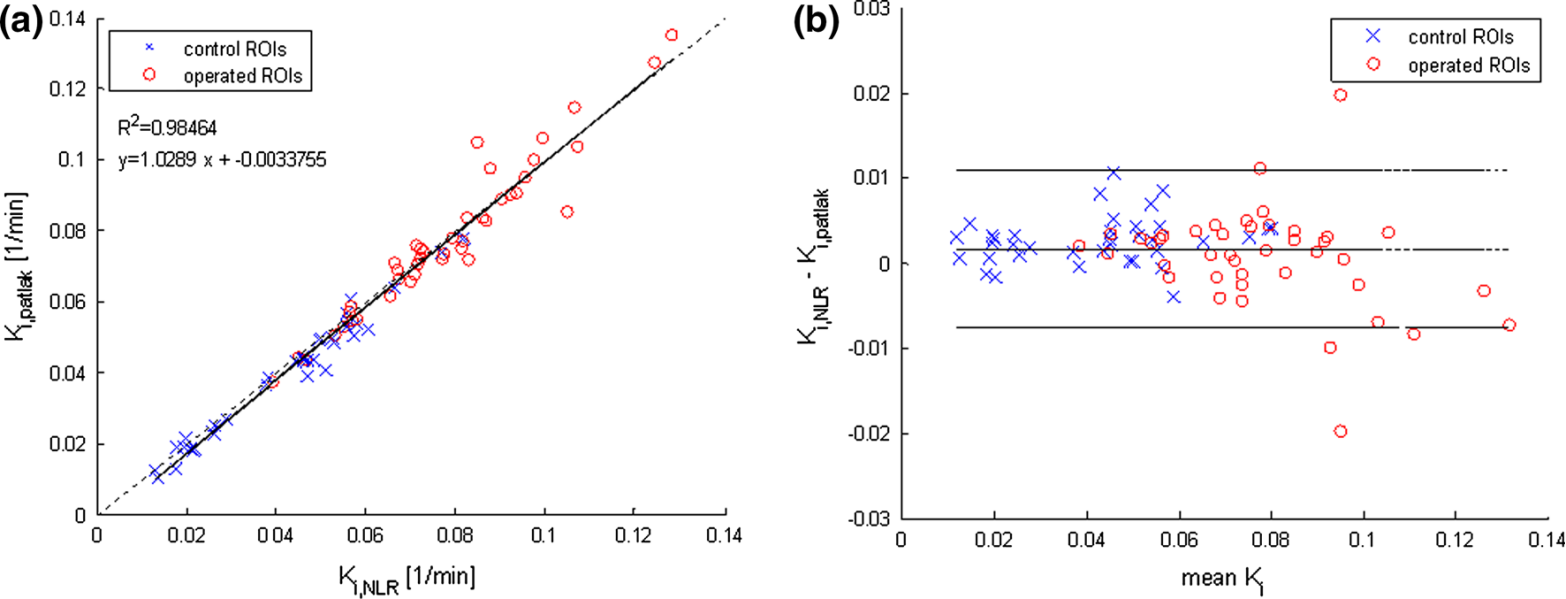
Correlation plot and Bland–Altman plot of K_*i*,NLR_ and *K*_i,patlak_. **a** Pearson correlation plot with correlation value *R* ^2^ (*p* < 0.0001) between *K*_i,NLR_ and *K*_i,patlak_. *Blue crosses* represent the control region data and *red* *circles* represent the operated region data. The *dotted line* in the graph is the line of identity. *Equation* *y* shows the deviation of the data from the line of identity, representing the difference between the two parameters. **b** Bland–Altman plot for the data shown in **a**. The *middle horizontal line* represents the mean difference value, the *upper* and *lower lines* represent the 95 % confidence interval

**Fig. 3:**
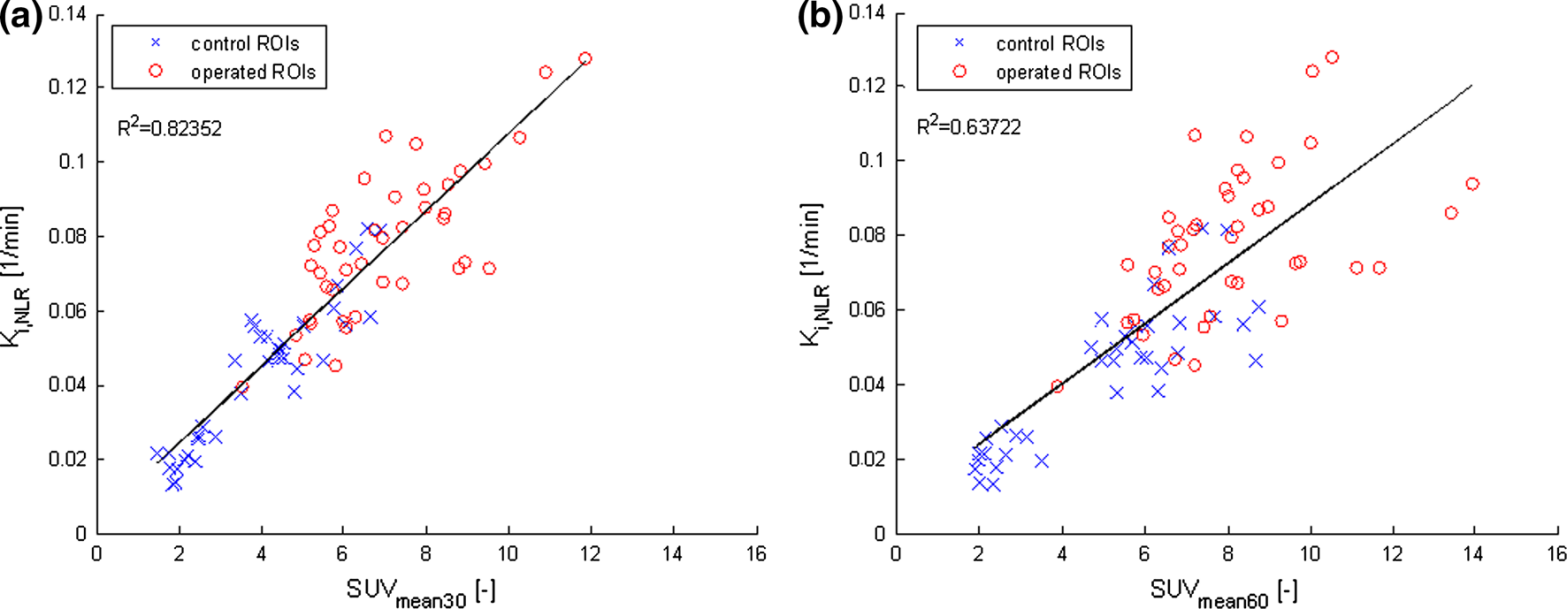
Correlation plots *K*_i,NLR_ and SUV_mean_ at 30 and 60 min. Pearson correlation plot with correlation values (*p* < 0.0001) between SUV_mean60_ (**a**), SUV_mean30_ (**b**) and *K*_i,NLR_. *Blue crosses* represent the control region data and *red circles* represent the operated region data

Figures 2 and 3 show the relationship between *K*_i,NLR_ and *K*_i,patlak_ and between SUV and *K*_i_ values, respectively. Figure 2a shows a very high correlation between *K*_i,NLR_ and *K*_i,patlak_ (Pearson correlation value of 0.98, *p* < 0.0001), indicating that the two dynamic approaches are robust. Figure 2b shows a Bland–Altman plot for the data shown in Fig. 2a. The mean difference between *K*_i,NLR_ and *K*_i,patlak_ was 0.0016 with 95 % limits of agreement of −0.0077 and +0.011. Moreover, the trend line almost coincided with the line of identity, indicating that *K*_i,NLR_ and *K*_i,patlak_ yield identical results. Figure 3 shows the correlation of the measured SUV_mean30_ (Fig. 3a) and SUV_mean60_ (Fig. 3b) with *K*_i,NLR_. Both SUV_mean30_ and SUV_mean60_ were highly correlated to *K*_i,NLR_ (*R*^2^ = 0.82 and *R*^2^ = 0.64, respectively). Despite this good correlation, it can be seen from the figures that for individual regions, rather large deviations from the trend line were present.

**Fig. 4:**
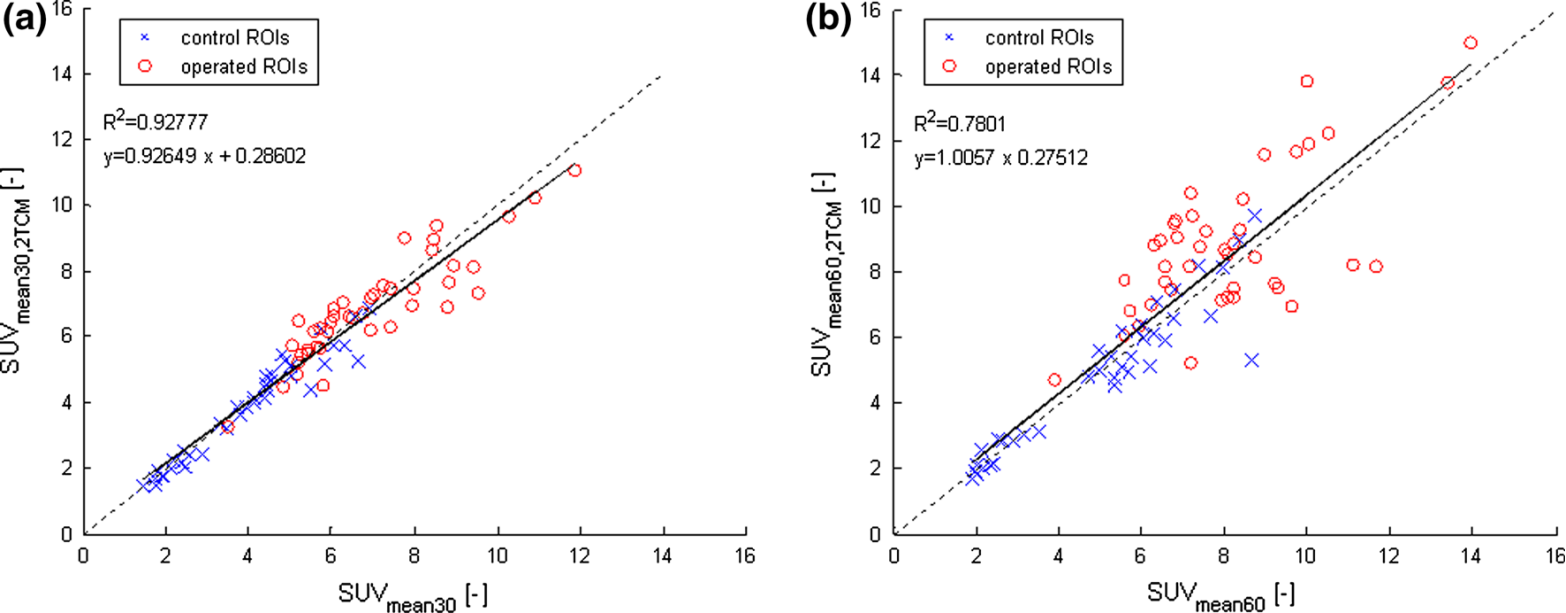
Correlation plots SUV_mean_ and SUV_mean,2TCM_ at 30 and 60 min. Pearson correlation plots with correlation values between the measured SUV_mean30_ and the calculated SUV_mean30,2TCM_ (**a**), and SUV_mean60_ and SUV_mean60,2TCM_ (**b**) (*p* < 0.0001). *Blue crosses* represent the control region data and *red circles* represent the operated region data. The *dotted line* in the graph is the line of identity. *Equation y* shows the deviation of the data from the line of identity, representing the difference between the two parameters

Figure 4 shows the relationship between the measured SUV_mean_ at 30 (Fig. 4a; SUV_mean30_) and 60 min (Fig. 4b; SUV_mean60_) with that of the calculated SUV_mean_ derived from the dynamic data (SUV_mean30/60,2TCM_). It can be observed that the deviation from the trend line decreased compared to Fig. 3, which was supported by an increase in *R*^2^ values to 0.93 and 0.78, respectively. With Eq. (3) in mind, this improvement in correlation after addition of $\tau^{*}$ indicated that the residence time was a patient-specific factor that added variability to SUV not directly related to local bone incorporation (*K*_i_).

**Fig. 5:**
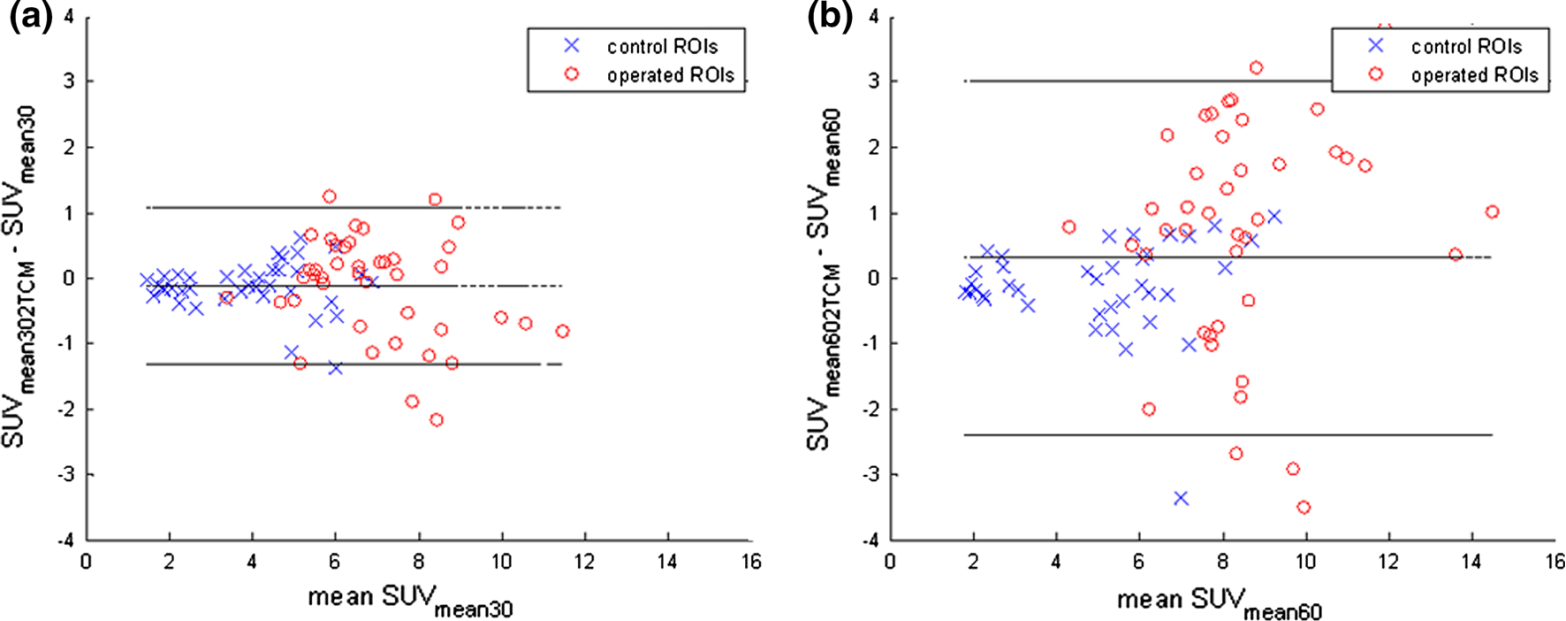
Bland–Altman plots of SUV_mean_ and SUV_mean,2TCM_ at 30 and 60 min. Bland–Altman plots, mean SUV measured from the PET scan (SUV_mean30_, SUV_mean60_) and calculated with *K*_i,NLR_ (SUV_mean30,2TCM_, SUV_mean60,2TCM_). The *middle horizontal line* represents the mean difference between SUV_mean_ and SUV_mean,2TCM_. The *upper* and *lower lines* represent the 95 % confidence interval limits. *Blue crosses* represent the control region data and *red circles* represent the operated region data

Figure 5 shows the Bland–Altman plots for the data shown in Fig. 4. The mean difference between SUV_mean30_ and SUV_mean30,2TCM_ was −0.052 with 95 % limits of agreement of −1.3 and +1.1. The mean difference between SUV_mean60_ and SUV_mean60,2TCM_ was +0.43 with larger 95 % limits of agreement of −2.3 and 3.0. In both Bland–Altman plots, only a few regions exceed the 95 % confidence interval (CI) limits. Using SUV_mean30,2TCM_, a measured SUV_mean30_ of 8 would be calculated from the dynamic data to lie between 7 and 9 with a CI of 95 %. Using SUV_mean60,2TCM_, a measured SUV_mean60_ of 8 would lie between 6 and 10.5 with a CI of 95 %. Thus, values of the calculated SUV_mean60,2TCM_ from the dynamic 30-min scan corresponded well with the measured SUV_mean60_ from the static scan at 60 min post-injection.

Table 2 shows the parameters in the pseudarthrosis (*n* = 6) and fused (*n* = 10) patient groups. Of all parameters obtained, only those regarding the incorporation of bone [*K*_i,NLR_, *K*_i,patlak_, *k*_3_/(*k*_2_ + *k*_3_)] differed statistically significant in the intervertebral disc space between the pseudarthrosis and fused patients group. The values of $\tau^{*}$ range between 64.4 and 115 Bq s/Bq with a standard deviation of 13.4 Bq s/Bq, which is 16.3 % of the mean value 81.9 Bq s/Bq. For the pseudarthrosis group, $\tau^{*}$ had a mean of 87.4 with a standard deviation of 7.2. For the fusion group, $\tau^{*}$ had a mean of 74.3 with a standard deviation of 7.4. The mean values of $\tau^{*}$ differed statistically significant between the pseudarthrosis and the fusion group, with a *p* value of 0.011. This may correspond with the lack of statistical significance of the SUV values.

**Table 2:** Parameter values in operated regions for pseudarthrosis and fusion patients

Parameter	Region of interest	Pseudarthrosis (*n* = 6)^a^	Fused (*n* = 10)^a^	*p* value^b^
SUV_mean30_ (–)	UE	8.5 (2.1)	7.2 (2.1)	0.279
	IDS	7.3 (2.4)	6.7 (1.8)	0.786
	LE	8.7 (2.1)	7.2 (1.6)	0.144
SUV_max30_ (–)	UE	13 (3.6)	10 (3.0)	0.100
	IDS	12 (3.5)	11 (3.0)	0.524
	LE	13 (3.5)	11 (2.8)	0.160
SUV_mean60_ (–)	UE	10 (3.2)	8.0 (1.4)	0.275
	IDS	7.3 (2.7)	7.5 (1.3)	0.622
	LE	11 (3.3)	8.0 (1.2)	0.126
SUV_max60_ (–)	UE	17 (5.8)	12 (2.3)	0.145
	IDS	14 (5.1)	12 (2.6)	0.617
	LE	17 (5.9)	12 (3.2)	0.170
*K*_i,patlak_ (min^−1^)	UE	0.077 (0.012)	0.082 (0.026)	0.551
	IDS	0.062 (0.018)	0.081 (0.020)	0.050
	LE	0.082 (0.017)	0.083 (0.022)	0.810
*K*_i,NLR_ (min^−1^)	UE	0.07 (0.013)	0.081 (0.021)	0.355
	IDS	0.045 (0.019)	0.083 (0.019)	0.004
	LE	0.07 (0.015)	0.084 (0.020)	0.137
*K*_1_ (ml g^−1^ min^−1^)	UE	0.19 (0.023)	0.19 (0.036)	0.937
	IDS	0.15 (0.038)	0.18 (0.045)	0.355
	LE	0.18 (0.023)	0.20 (0.090)	0.516
*k*_2_ (min^−1^)	UE	0.19 (0.11)	0.25 (0.24)	0.333
	IDS	0.22 (0.16)	0.29 (0.28)	0.454
	LE	0.16 (0.094)	0.39 (0.75)	0.448
*k*_3_ (min^−1^)	UE	0.10 (0.045)	0.17 (0.15)	0.266
	IDS	0.09 (0.063)	0.22 (0.18)	0.066
	LE	0.10 (0.055)	0.19 (0.18)	0.269
*v*_b_ (–)	UE	0.047 (0.019)	0.037 (0.012)	0.152
	IDS	0.046 (0.020)	0.037 (0.013)	0.547
	LE	0.046 (0.020)	0.036 (0.013)	0.431
*K*_1_/*k*_2_ (ml g^−1^)	UE	1.8 (1.9)	2.1 (2.5)	0.894
	IDS	0.6 (2.1)	1.6 (1.8)	0.879
	LE	1.9 (1.9)	2.9 (5.6)	0.759
*k*_3_/(k_*2*_ + *k*_3_) (–)	UE	0.36 (0.044)	0.44 (0.12)	0.215
	IDS	0.29 (0.091)	0.47 (0.13)	0.016
	LE	0.40 (0.067)	0.46 (0.18)	0.506

*UE* upper endplate, *IDS* intervertebral disc space, *LE* lower endplate

^a^Values as mean (SD in brackets), 2 significant digits

^b^The *p* value indicates the statistical difference between the pseudarthrosis and fusion patients

## Discussion

Blake has shown that the dependency of SUV on clearance rate can lead to erroneous conclusions as opposed to *K*_i_ which is independent of clearance rate [22]. Small differences were only detectable with *K*_i_ [30, 31]. Brenner found, in a study on limb and thoracic spine bone grafts, that with *K*_i_, smaller changes in bone metabolism could be detected than with SUV due to a wider 95 % range of results for SUV (±58.0 %) as compared to *K*_i,NLR_ (±20.2 %) and *K*_i,patlak_ (±23.0 %) [31]. Although the patient population in these studies might not be representative for our present population, it shows the effect that differences in clearance can have on results. In our study, the rate of clearance was incorporated in the parameter $\tau^{*}$. It was shown that in this particular patient population, the patient-specific factor $\tau^{*}$ introduced an additional inter-subject variability of 16.3 % to SUV that was not directly related to local bone metabolism. Table 2 shows significant differences between patient groups in dynamic parameters *K*_i,NLR_, *K*_i,patlak_ and *k*_3_/(*k*_2_ + *k*_3_) but not in static parameters (SUV). This can be explained by the significant difference in $\tau^{*}$ between patient groups, which is accounted for in dynamic but not in static analysis. Our results suggest that blood clearance rates in patients suffering from pseudarthrosis is altered and thus dynamic parameters are of possible additional value in the evaluation of such patients. However, further prospective studies in larger and more homogeneous patient groups must be done to confirm these results.

The dynamic parameter *K*_1_ has experimentally been shown to relate to blood bone perfusion in a porcine model [32] and used by others in the study of patients with hip and lumbar spine osteoporosis [27]. Recently, in a small study in patients comparing mandibular or hip surgery patients with normal volunteers, Raijmakers et al. [33] showed a low correlation between K_1_ and bone blood flow as measured by ^15^O–H_2_O PET. Puri suggested that changes in *k*_3_/(*k*_2_ + *k*_3_) may be the best means of using ^18^F-PET scans to investigate changes in osteoblastic activity [27]. In our study, the parameters *K*_1_, *k*_2_, *k*_3_ and *v*_b_ were not statistically significant different between all control and operated regions. This observation might correspond to the reported decrease of stability of *K*_1_, *k*_2_, *k*_3_ compared to *K*_i_,_NLR_ [31, 34, 35]. Cook et al. stated that due to possible limitations of the model, the physiological significance of parameters *k*_2_ and *k*_3_ is not meaningful in relation to the mineralized skeleton [35]. Our study suggests that *k*_3_/(*k*_2_ + *k*_3_), the parameter that represents the portion ^18^F-fluoride that binds to the mineral after entering the unbound compartment, is also a stable parameter with statistical significance between control and operated ROIs of 0.0030–0.016 in Table 1 as well as in the intervertebral disc space between pseudarthrosis and fused patients (*p* = 0.016) in Table 2. *K*_1_/*k*_2_ could be interesting as well with a large effect size and statistical significance between control and operated ROIs of 0.0040–0.030 in Table 1. The ability to separately evaluate *K*_1_, *K*_1_/*k*_2_ and *k*_3_/(*k*_2_ + *k*_3_) allows one to distinguish between different biological processes in a ROI. Separate parameters related to blood flow and to osteoblastic activity can be of great value in the early assessment of patients after PLIF surgery. Future studies have to show whether these differences in dynamic parameters are clinically relevant in particular patient groups other than the spinal surgery patients used in this study.

The study has a number of limitations. Dynamic scanning was performed for only 30 min, while several other ^18^F-fluoride dynamic studies scanned for 60 min [22, 33, 36]. However, such a long protocol was not feasible in these patients with considerable degree of back pain. Since our scan protocol included a dynamic and a static scan in between which the patient left the scanner, the regions drawn in the dynamic and static scan did not coincide perfectly, which influenced the observed difference in correlation coefficient of *K*_i,NLR_ and SUV_mean60_ as compared to *K*_i,NLR_ and SUV_mean30_. This also resulted in a larger 95 % CI in the Bland–Altman plot of SUV_mean60_ and SUV_mean60,2TCM_ as compared to the plot of SUV_mean30_ and SUV_mean30,2TCM_. Therefore, *K*_i,NLR_ was also compared to SUV at 30 min since in this comparison the exact same ROIs were used. However, our objective was not to compare both time intervals, but to test the feasibility of a relatively short image acquisition in view of patient comfort, clinical applicability and patient throughput. Indeed, given the rapid kinetics of ^18^F-fluoride [37], other authors [21] have previously shown that it is possible to estimate *K*_i,NLR_ from a 4-min static scan of the lumbar spine between 30 and 60 min together with 2–4 venous blood samples, providing errors relative to the Patlak values of +0.6 % at 30 min after injection, increasing up to −3.3 % at 60 min. Although this is a different clinical approach, the present results show correlations of parameters similar to values reported in other studies, indicating that dynamic scanning for 30 min may be sufficient. Furthermore, the time interval between PLIF surgery and PET/CT was variable. However, inclusion was performed on the basis of clinical ground, i.e., persistent back pain after lumbar surgery. Besides, for the aim of this study, to compare the static and dynamic analysis methods, this was not an issue. Our analysis approach involved the use of an IDIF instead of the gold standard of arterial sampling. The use of an IDIF in the aorta to obtain bone metabolism values in the spine has been performed before and was validated against arterial sampling [38].

To conclude, this study shows the feasibility of a 30 min dynamic ^18^F-fluoride PET/CT scanning and this may provide dynamic parameters clinically relevant to the diagnosis of pseudarthrosis.

## Appendix: Analytical solution for time-dependent tissue radioactivity concentration

**Fig. 6:**
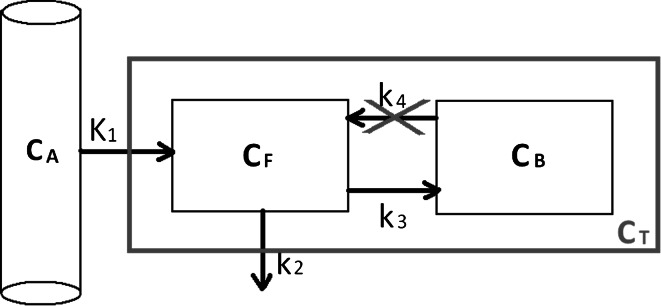
The 2TCM model. Three-compartment, 4-parameter model for fluoride bone metabolism. Each compartment contains a certain concentration of ^18^F: *C*_A_ (Bq/ml) is the arterial radioactivity concentration, *C*_F_ (Bq/ml) represents the free concentration in the extravascular space unbound to bone and *C*_B_ (Bq/ml) refers to the radioactivity bound to bone either on the bone surface or fully incorporated in the hydroxyapatite [23]. *C*_T_ (Bq/ml) represents the total tissue radioactivity concentration that the PET scanner will measure, which is equal to the summation of *C*_F_ and *C*_B_. The rate constants *K*_1_–*k*_4_ describe the transport rate of the fluoride between the compartments. Rate constant *k*_4_ can be ignored for this particular situation since the amount of fluoride that will be released after binding to the hydroxyapatite within the time frame of the PET measurement is negligible. A fractional blood volume parameter, *v*_b_ (–), was also included in the model to account for the plasma and red cell ^18^F-fluoride activity in the tissue region. Due to gains and losses from adjacent compartments, the rate of change of tracer concentration in the extravascular space and in the bound compartment can be described

NLR analysis was based on the irreversible 2TCM of Hawkins [23] with three parameters and a blood volume fraction (Fig. 6).

The measured radioactivity concentration can be calculated from the dynamic parameters, since *C*_T_ in Fig. 6 represents the radioactivity concentration measured by the PET scan. Solving the system of differential Eq. (4) yields expressions for the radioactivity concentration in the compartments *C*_F_ and *C*_B_ which can be combined into an expression for *C*_T_ (5) that can deduced from Fig. 6.

4 $$\left\{ \begin{aligned} \frac{{{\text{d}}C_{\text{F}} (t)}}{{{\text{d}}t}} & = K_{1} C_{\text{A}} (t) - \left( {k_{2} + k_{3} } \right)C_{\text{F}} (t) \\ \frac{{{\text{d}}C_{\text{B}} (t)}}{{{\text{d}}t}} & = k_{3} C_{\text{F}} (t) \\ \end{aligned} \right.$$

5 $$C_{T} \left( t \right) = C_{F} \left( t \right) + C_{B} \left( t \right) = [K_{1} \times e^{{ - \left( {k_{2} + k_{3} } \right)t}} + K_{i,NLR} \left( {1 - e^{{ - (k_{2} + k_{3} )t}} } \right)] \otimes C_{A} (t)$$

Correcting *C*_T_ for the body weight and injected dose yields SUV_exact_ (6).

6 $${\text{SUV}}_{\text{exact}} = \frac{{C_{\text{T}} (t)}}{{{\text{ID}}/m}} = \frac{{[K_{1} \times e^{{ - \left( {k_{2} + k_{3} } \right)t}} + K_{\text{i, NLR}} \left( {1 - e^{{ - (k_{2} + k_{3} )t}} } \right)] \otimes C_{\text{A}} (t)}}{{{\text{ID}}/m}}$$

When *t* approaches infinity, this expression can be simplified to (7).

7 $$\mathop {\lim }\limits_{t \to \infty } {\text{SUV}}_{{ 2 {\text{TCM}}}} \approx \frac{{K_{\text{i, NLR}} \times \mathop \smallint \nolimits_{0}^{t} C_{\text{A}} (t){\text{d}}t}}{{{\text{ID}}/m}}$$

Whether the exact relation (6) or the simplification (7) was used to calculate SUV_mean,2TCM_ is dependent on whether the assumption of time goes to infinity applied to the particular time point.

The arterial input concentration (*C*_A_) is known for the first 30 min from the dynamic scan. To obtain the arterial input concentration at later time points, the *C*_A_ curve was extrapolated with an exponential function from the peak of the *C*_A_ curve of 0–30 min.

The factor between SUV and *K*_i,NLR_ can also be written as physically interpretable terms (8).

8 $$\mathop {\lim }\limits_{t \to \infty } {\text{SUV}}_{{ 2 {\text{TCM}}}} = K_{\text{i, NLR}} \times \frac{{\mathop \smallint \nolimits A\left( t \right){\text{d}}t}}{{A_{0} }} \times \frac{m}{{V_{\text{b}} }} = K_{\text{i, NLR}} \times \tau \times \frac{1}{{f_{\text{b}} }} = K_{\text{i, NLR}} \times \tau$$
